# Stabilization Beyond Mood: Stabilizing Patients With Bipolar Disorder in the Various Phases of Life

**DOI:** 10.3389/fpsyt.2020.00247

**Published:** 2020-04-27

**Authors:** Alessio Simonetti, Alexia E. Koukopoulos, Georgios D. Kotzalidis, Delfina Janiri, Lavinia De Chiara, Luigi Janiri, Gabriele Sani

**Affiliations:** ^1^Menninger Department of Psychiatry and Behavioral Sciences, Baylor College of Medicine, Houston, TX, United States; ^2^Department of Neurology and Psychiatry, Sapienza University of Rome, Rome, Italy; ^3^Centro Lucio Bini, Rome, Italy; ^4^Azienda Ospedaliera Universitaria Policlinico Umberto I, Sapienza School of Medicine and Dentistry, Sapienza University of Rome, Rome, Italy; ^5^NESMOS Department, Faculty of Medicine and Psychology, Sapienza University of Rome, Sant'Andrea University Hospital, Rome, Italy; ^6^Institute of Psychiatry, Università Cattolica del Sacro Cuore, Rome, Italy; ^7^Department of Psychiatry, Fondazione Policlinico Universitario “Agostino Gemelli” IRCCS, Rome, Italy

**Keywords:** mood, stabilization, antidepressant drugs, antipsychotic drugs, lithium, mood stabilizers

## Abstract

**Background:**

There are different ways to define stabilization and currently, the main standpoint regards it as no-depression/no-mania. Furthermore, each person is physiologically different from childhood to adulthood, and in old age, thus the meaning of stabilization should take into account both growth and maturity. We aimed to review systematically studies focusing on mood stabilization in all phases of bipolar disorder (BD) and across all life phases, including pregnancy and the perinatal period, which is still a different phase in women's life cycles.

**Methods:**

We carried out a PubMed search focusing on studies of bipolar disorder treated with drugs and aimed at stabilization with the following search strategy stabiliz*[ti] OR stabilis*[ti] OR stable[ti] OR stability[ti]) AND mood[ti] AND bipolar. In conducting our review, we followed the *PRISMA* statement. Agreement on inclusion was reached by consensus of all authors through a Delphi rounds procedure.

**Results:**

The above search strategy produced 509 records on January 25, 2020. Of them, 58 fitted our inclusion criteria and were discussed. The eligible studies spanned from September 1983 to July 6, 2019.

**Conclusions:**

No clear-cut indications could be drawn due to a number of limitations involving sample inconsistency and different methods of assessing mood stabilization. The evidence collected so far does not allow recommended treatments for Adolescents, pregnant or perinatal women, and aged patients. However, adults, not within these groups, better focused upon. For their manic/mixed phases, second generation antipsychotic drugs may be useful in the short-to-medium run, alone or combined with mood stabilizers (MSs). However, MSs, and especially lithium, continue to be pivotal in chronic treatment. Bipolar depression should rely on MSs, but an antidepressant may be added on and can prove to be helpful. However, there are concerns with the tendency of antidepressants to induce the opposite polarity or mood instability, rendering the need for concurrent MS prescription mandatory.

## Introduction

The treatment of bipolar disorder (BD) is currently unsatisfactory. Despite the good results obtained in the treatment of its acute manic phase, this may also be the result of the natural course of the disorder. The challenge would be to obtain a clinical response that maintains patients euthymic, without mood swings, and for a sustained time-period. The latter is unfortunately an unmet need, because few patients manage to stay in treatment for a sufficient time to be declared as remitted. In fact, 40% (1) to 60% (2) of patients discontinue lithium after 5-7 years, and despite good adherence, some 13% of patients who were responders for five years, become resistant to lithium treatment after 10 years (3). BD has its onset usually in late adolescence and early adulthood, less often in later adulthood or advanced age, and seldom during childhood (4), with each range of age at onset displaying a normal-like distribution (5). Since it runs a cyclic course, with manic and depressive episodes and with relatively asymptomatic intervals, and is a biologically heterogeneous entity with precise neural (6–9) and peripheral correlates (10), BD needs to be treated according to subtype and for the entire life span (11). Drug treatment of BD is further complicated not only by the side effects that keep patients away from treatment, but also by long-term drug-induced alterations that prompt doctors to stop or switch to other drugs. For example, there is much concern about the long-term nephrotoxicity of lithium (12–14), but other mood stabilizers (MSs) are not devoid of dangerous side effects (15). Hence, drug treatment of BD has to be tailor-cut to patient's individual needs (16).

Different concepts are included in what we mean by mood stabilization. Mood is normally swaying between what is not mania and not depression, something we call euthymia, but is not a flat line. Consequently, an optimal MS should keep the patient within this range. However, hypomania, which is subsyndromal with respect to mania, is not an acceptable state, since it is often linked to BD-II type and is likely to be followed by bipolar depression, which is a clinical state most unpleasant to the patients and their doctors. It is generally accepted that to be called a MS, a drug must relieve at least one phase of BD and not to cause the opposite. However, this simplistic definition would include antidepressant (ADs) and antipsychotic drugs (APs) as well, not causing respectively (hypo)mania or depression, adding much to the confusion. There is much debate about how much ADs trigger manic switches and how some second-generation APs are endowed with antidepressant action and currently indicated by the US Food and Drug Administration (FDA) for depression (for example, lurasidone and brexpiprazole). This prompted Nassir Ghaemi (17) to develop an elaborate concept of MS, proposing that a MS should be conservatively defined as “an agent with efficacy in two of the three phases of bipolar illness (acute depression, acute mania, prophylaxis)”. In this definition, prophylaxis is meant as a protection from the occurrence of either manic or depressive episodes. This definition excludes all APs and leaves lithium, carbamazepine, valproate, and lamotrigine. Terence Ketter's proposal many years later (18), retained much of the essence of Ghaemi's proposed definition, by stating “any treatment that is effective in any phase of bipolar disorder (an America‐centric approach would be to say FDA‐approved for any phase of bipolar disorder) but not active at dopamine receptors (thus excluding antipsychotics)”. He should have added directly at *dopamine* receptors, since lithium and other MSs indirectly affect dopaminergic transmission (19–21). Both these definitions are acceptable and the drugs they envisage as MSs are the ones we will here consider. The identification of a neurochemical signature of mood stabilization, like a decreased glutamate-to-gamma aminobutyric acid ratio or genetic markers such as the GAD1 rs1978340 allele A (22), would greatly aid and steer the future pharmacological treatment strategies. However, their adoption in treatment models of BD should await confirmation by future studies (and the identification of other markers as well is to be expected.

The onset of BD during infancy is an extremely rare event. However, in many instances, it develops during adolescence. The latter is a period of rapid physiological changes and adaptation, with the brain in continuous maturation. It is accepted that the brain does not conclude its developmental trajectory before the 24^th^ year of life (23). Any action of a drug at this stage might affect further development, hence particular caution is mandatory in facing cases of adolescent BD. Furthermore, the brain in the two genders matures differently, both in normal (24) and BD adolescents (25), thus forcing treating clinicians to personalize their interventions by taking into account multiple factors, including gender, and substance use that could arrest a normal maturational process in the neurobiological interplay between the “inbuilt” underlying disorder and the “acquired” substance use disorder (26). The gender concern comes to the fore when women become pregnant. During this particular phase of life, the hormonal turmoil that occurs during gestation and the post-partum makes the woman vulnerable to psychiatric events, including later first occurrences or recurrences of BD (27, 28). The old age comes with a decay of functioning bodily systems, including the brain, so the clinical expression of BD and the organism's response to drugs are consequently affected. Generally, dose adjustments of the same types of medications are sufficient to deal with BD in the elderly.

### Aim of the Review

To identify drug treatment patterns for BD stabilization across different phases of life, we conducted a systematic review with keywords focusing on mood stabilization and bipolar disorder. The studies emerging from database search were subsequently subdivided according to the age range involved or the special condition (pregnancy or postpartum).

## Methods

We conducted our review according to the *P*referred *R*eporting *I*tems for *S*ystematic reviews and *M*eta-*A*nalyses (*PRISMA*) statement (29). We search the PubMed database using the following strategy: stabiliz*[ti] OR stabilis*[ti] OR stable[ti] OR stability[ti]) AND mood[ti] AND bipolar. Papers were individually searched for adherence to our inclusion criteria. Retrieved relevant papers, comprising reviews and meta-analyses, were searched in their reference lists for providing additional papers with adequate research data and meeting our inclusion criteria. These were: double blind, placebo or comparator-controlled trials, open-label trials and naturalistic studies investigating the efficacy/effectiveness of drugs given either as monotherapy or add-on medication and belonging to drug classes like MSs, ADs, first generation APs (FGAs), second-generation APs (SGAs), anticonvulsant benzodiazepines (BDZs), or nonconventional mood-stabilizing medications (e.g., allopurinol) in reducing manic, hypomanic, mixed, or depressive symptoms and/or preventing the occurrence of new mood episodes in patients with BD.

Exclusion criteria were: reviews and meta-analyses, animal and *in vitro* studies, unfocused studies, i.e., studies with nonclinical outcomes not reporting efficacy data, editorials and opinion papers, like letters to the editor with no data or comments on other literature, case reports and case series with no reliable statistics, studies not focusing on BD or including disorders other than BD without separately reporting on BD, congress/conference abstracts, studies lacking clinical data, studies focusing on pharmacokinetics, surveys, studies of registries, papers reporting on data originally published by others. When a study was extended and used the same sample on which results were previously reported, we eliminated the first report and kept the paper with the larger sample, provided that quality of reporting was maintained. We accurately avoided to include studies referring to the same patient sample.

Inclusion and exclusion were based on consensus among all authors; unanimity was required for both and was achieved through Delphi rounds. Two rounds were sufficient to reach complete agreement among authors.

## Results

**Figure 1:**
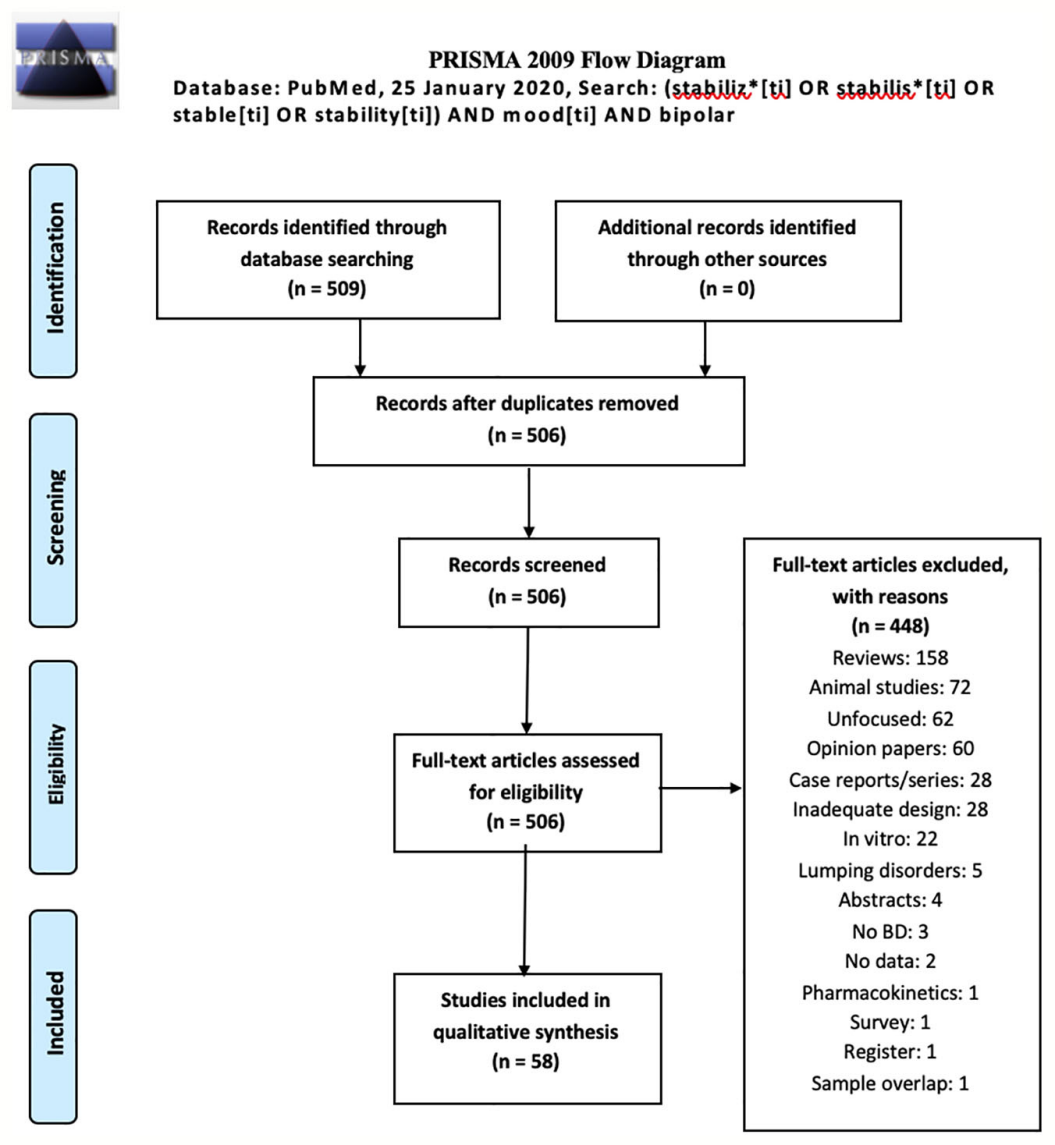
*PRISMA* flowchart of our review's results (30).

Our search produced 509 records on January 25, 2020. Authors identified 3 duplicates, which were excluded; hence, the pooled records amounted to 506. Excluded were: 158 reviews, 72 animal studies, 62 unfocused studies, 60 opinion/editorial papers, 28 case reports/case series, 28 studies with inadequate design, 22 *in vitro* (nonanimal) studies, 5 studies on mixed samples which did not provide data for subgroups affected by BD (identified as lumping), 4 congress/conference abstracts without complete data, 3 studies not focusing on BD samples, identified as no BD, 2 studies lacking clinical data, 1 study on pharmacokinetics, 1 survey, 1 registry study, 1 study which reported data originally published by others (sample overlap). Therefore, the final number of studies included in this review was 58. The results of our search is shown as a *PRISMA* flowchart in **Figure 1** with the reasons of exclusion. Detailed, study per study information about inclusion/exclusion is provided in the supplement. The search of reference lists of reviews yielded no further articles.

Included studies are summarized in **Table 1**. They spanned from September 1983 to July 6, 2019, while the complete output spanned from April 1970 to December 19, 2019. We split our included records as childhood and adolescence (*N*=3), adulthood (*N*=52), old age (*N*=2), and pregnancy/perinatal period (*N*=1). One study was conducted on both elderly and adult patients. Since all nonadult trials were few, we applied a further distinction on studies of adults, subdivided into acute phase (*N*=31), single drug (*N*=3), add-on (*N*=25) or mixed (*N*=3), and long-term with survival curves (Kaplan-Meier) (*N*=10). All articles were in English, in spite of the fact that non-English language was not an exclusionary criterion.

**Table 1:** Summary of included studies.

Study	Population	Design	Results	Conclusions
31	17 euthymic BD-I on Li+ × 35±37 months (7 ♂; 10 ♀) $\bar{x}$=37±10 *vs.* 21 HCs (7 ♂; 14 ♀) $\bar{x}$=30±8 (the latter were significantly younger, *p*<0.05)	Self-rated mood stability of pts. with BD and HCs with VAMS × 11 days	Patients on Li^+^ did not differ from HCs on VAMS ratings, but rated themselves as less swinging than HCs (6.9±4 *vs.* 12.5±6; *p*<0.01); two patients withdrawn from Li+ developed a manic episode; another did not, but developed mood instability	Li^+^ confers mood stabilization independently from age or gender
32	120 BDI, aged >60 ($\bar{x}$=40.3±15.3; 52 ♂ and 68 ♀). Two groups: manic (N=50); mixed (N=70), further divided in those who have received Li^+^, AEs, or both	Naturalistic, retrospective (length of hospital stay). Outcome measures: between-group differences; Remission criteria: CGI-I score ≥2	Serum VPA levels were higher in mixed (84.7 ± 13.9) than manic (64.6 ± 12.8) group. In mixed, pts. receiving AE+Li^+^ have a slower remission than those receiving Li^+^ alone or AE alone. Many pts. on Li^+^+AE had the AE added after Li^+^ proved ineffective. After adding AE to Li^+^, they achieved remission in 2-3 weeks. In the whole pt. group, weeks needed to achieve adequate MS serum level was a predictor of remission	Effectiveness of Li^+^ and AEs in acute treatment of mixed or pure mania are similar. Time course to remission seems to be influenced by the speed with which patients achieve a therapeutic serum MS levels
33	72 BD inpts. aged >60 (mean age 67.19 ± 5.34); 27 ♂ and 45 *♀*	Retrospective chart review; Pts. were maintained on MS monotherapy with Li^+^ (41), CBZ (11), or VPA (20) during hospitalization; Outcome measures: length of treatment and change in GAF scores	Length of hospital stay was 27.5±15.4 days for pts. on CBZ, 22.1±11.2 days for pts. on Li+, and 24.8±13.5 days for pts. on VPA. ↑GAF 28.8±11.8 for pts. on CBZ, 29.9±15.8 for pts. on Li^+^, and 35.2±10.9 for pts. on VPA	No significant differences in outcome measures of acutely ill geriatric pts. with BD who were treated with Li^+^, VPA, or CBZ alone
34	10 BD-I, mixed, aged 37-72 ($\bar{x}$=50.4±2.8; 9 ♂; 1 ♀)	Open, ad- on gabapentin 900 2700 mg/day × 4 weeks; outcome measures: reduction of HDRS and BRMaS	Add on gabapentin ↓ HDRS (15 8) and BRMaS scores (8 1) at week 1. BRMaS scores stabilized through week 4; HDRS scores continued to ↓ (8 4)	Gabapentin potentiates the effect of the other mood stabilizers in subjects with BD and a mixed state
35	27 BD, depressed, aged 37-72 ($\bar{x}$=41±12; 9 ♂; 18 ♀, 11 BD-I, 16 BD-II). Two consecutive HDRS>16	DB, add-on paroxetine (36 mg/day) or additional MS (Li^+^, 1300 mg/day; VPA 1200 mg/day) Li^+^, in pts. already treated with an MS (Li^+^ or VPA, same dosages as above) × 6 weeks. Outcome measures: HDRS; YMRS; GAF scores	After 6 weeks, significant ↓ in HDRS scores in both groups, with no differences between groups. YMRS unchanged in both groups	Both adding another MS and adding an AD are effective in treating BD depression
36	31 adults with DSM-IV BD, last episode mixed	Open add-on risperidone $\bar{x}$=4.2 mg/day on ongoing mood stabilizer × 6 months, weekly assessed with YMRS, CGI, PANSS, and HDRS. Response criteria: YMRS ≥50%↓from BL AND CGI ≥2↓from BL	26 completers (84%); 74% responders at week 4; significant improvement on all scales, from week 1 onward on YMRS and HDRS, and from week 4 on CGI and PANSS; 73% asymptomatic at 6 months	Risperidone effective as add-on, but results need replication
37	158 depressed BD-I inpts. ($\bar{x}$ age=52.6±15; 50♂; 108♀)	Retrospective, naturalistic, evaluation of the impact of AD treatment on switch incidence from depression to mania/hypomania	25% switches; TCAs associated with higher risk of switching, reduced risk if ADs+MS	Better add AD only when MS is established
38	11 BD, aged 19 – 46 ($\bar{x}$=29.4±10.7; 10♂; 1♀; 6 BD-I, 4 BD-II)	Open, add-on vitamin and mineral capsules (36 supplements) ×≥6 weeks (range: 6-21 weeks). Outcome measures: reduction in HDRS, YMRS, and BPRS scores; Response criteria (≥20% improvement on all scales)	As compared with the first assessment, at last assessment, HDRS decrement was 55% and YMRS decrement was 66%. Rates of response were 87.5%. Number of standard medications/pt ↓ from 2.7±2.0 to 1.0±1.1	Vitamin and mineral capsules have beneficial psychotropic effects
39	155, BDI, manic; 4 groups: MS + atypical AP (RISP or OLZ, N=69, $\bar{x}$=39.72 ± 14.50 11 BD, aged 19 – 46 ($\bar{x}$=29.4±10.7; 32♂; 37♀); MS + typical AP (N=69, $\bar{x}$=40.86 ± 16.11, 37♂; 32♀); MS + combination of typical and atypical (first typical than switch with atypical, N= 17, 41.06 ± 18.08, 8♂; 9♀)	Naturalistic, retrospective; outcome measures: length of stay, CGI improvement	No differences in length of stay were found. At discharge, subjects with MS + atypical AP and subjects with MS + combination between typical and atypical AP showed smaller CGI improvement scores (1.59±.58; 1.56±.63 respectively) than those with MS + typical AP (2.04±.73). Same results when subgroups showing psychotic features were selected. Subgroup of subjects treated with MS + RISP showed greater CGI score improvement than those treated with MS + typical AP	SGAs, in particular RISP, might be more effective than typical APs combined with MS, to treat manic episodes. If pts. require initial treatment with MS+FGA, they might have a better outcome if they switch to an SGA after the 1^st^ week of treatment
40	64 depressive pts. (IDS≥16) while on mood stabilizers, aged 22.5-75,3 yrs ($\bar{x}$= 44.8±11.8; 38 ♂; 26 *♀*; 4*3* BD-I, 19 BD-II; 1 BD NOS, 1 SABT)	5-site DB; randomization to add-on bupropion 100 450 mg/day, sertraline 50 200 mg/day, or venlafaxine 75 375 mg/day, on ongoii8yhoy po60nk0long mood stabilizers × 10 weeks; nonresponders, rerandomized to other AD × 10 weeks, if still nonresponders, to the third; 1-year continuation on what works; Assessment with CGI-BP, response criterion, score 1 or 2	21 nonresponders rerandomized, 10 of them further rerandomized; a total of 95 acute-phase treatments were available for outcome assessment. 35 pts. exposed to acute phase treatment were responding (37%). During the 95 acute AD-exposure phases, there were 13 (14%) switches to mania/hypomania	Some depressions subsided and some switched to mania, but conclusive considerations could not be made due to the peculiarities of the design. Furthermore, final results were not available and 1 site could not administer bupropion
41	19 depressed outpts. BD-II ($\bar{x}$ age=29; 13 ♂; 6 ♀)	12-weeks open, divalproex sodium single dose 250 mg increased by 250 mg every 4 days until symptom relief in medication naïve *vs.* MS naïve; response criterion, ≥50% ↓ in HDRS scores	63% responders; higher response in medication naïve group	Results support divalproex sodium monotherapy in BD-II depression
42	156 BD-I, manic/mixed, aged 18-65 yrs. Three groups: RISP+MS (N=52; median=41 yrs; 26 ♂; 26 ♀); HAL+MS (N=53; median=44 yrs; 30 ♂; 23 ♀); placebo + MS (N=51; median=43 yrs; 24 ♂; 27 ♀)	3-week DB, placebo-controlled trial of RISP (range 2-6 mg)+MS (VPA: 65.4±27.1 µg/ml; Li^+^: 0.7±0.3 meq/l) and HAL (4-12 mg)+MS (VPA: 76.2±25.6; Li^+^: 0.7±0.2 meq/l). Outcome measures: changes in YMRS scores, % of pts. scoring 1 on the CGI-I (“very much improved”)	RISP+MS and HAL+MS lowered YMRS scores more than placebo+MS (-14.3 *vs.* -13.4 *vs.* -8.2, respectively). RISP+MS and HAL+MS obtained higher rates of “very much improved” on the CGI-I than placebo+MS (50% *vs.* 53% *vs.* 30%, respectively). No between-group differences in psychotic *vs.* nonpsychotic and manic *vs.* mixed subpopulations. The HAL+MS group worsened more than placebo+MS scores on the Extrapyramidal Symptom Rating Scale from BL to endpoint (2 *vs.* -0.1, respectively, and maximum score 1.9 *vs.* 5.4)	Both HAL and RISP are effective adjunctive treatments for manic and mixed states. RISP has a safer profile
43	115 BD-I, manic or mixed, aged 18-70 yrs (*$\bar{x}$*=39yrs; 60♂; 58♀)	DB, placebo-controlled trial of OLZ (5-20 mg/day)+MS (Li^+^, VPA). Each group further divided in MS nonresponders *vs.* other (responders or those who were not exposed to MSs before randomization (MS-naïve). Outcome measures: YMRS score ↓, percentage of remission	YMRS ↓ more in the OLZ+MS than in the OLZ+placebo group. Previous response to VPA or Li^+^ does not affect results (YMRS ↓ in OLZ+VPA responders *vs.* OLZ+VPA-others: -14.7 *vs.* -14.8; YMRS ↓ in placebo+VPA responders *vs.* placebo+VPA-others: -8.8 *vs.* -8.0; YMRS ↓ in OLZ+Li^+^ responders *vs.* OLZ+Li^+^-others: -15.9 *vs.* -13.9; YMRS ↓ in placebo+ Li^+^-responders *vs.* placebo+Li^+^-others: -6.5 *vs.* -8.9; proportion of pts. who remitted in OLZ+VPA responders *vs.* OLZ+VPA-others: 61.9% *vs.* 60.6%; proportion of pts. who remitted in placebo+VPA responders *vs.* placebo+VPA-others: 40.0% *vs.* 34.8%; proportion of pts. who remitted in OLZ+Li^+^ responders *vs.* OLZ+Li^+^-others: 66.7% *vs.* 56.7%; YMRS score ↓ in placebo+Li^+^-responders *vs.* placebo+Li^+^-others: 33.3% *vs.* 36.8%	OLZ was superior to placebo in treating mania. This secondary analysis suggests that OLZ monotherapy is similarly effective for pts. whether or not they have previously failed to respond to another MS for mania
44	36 BD depressive, aged 18-70. Two groups: TPX+MS+SGAs (*N*=18; *$\bar{x}$*=39 yrs; 11 ♂; 7 ♀;10 BD-I, 8 BD-II); and bupropion+MS+SGAs (*N*=18; *$\bar{x}$*=43 yrs; 10 ♂; 8 ♀; 9 BD-I, 9 BD-II)	8-week, single blind add-on therapy with TPX (50-100 mg/day), or bupropion (100-400 mg/day) to MS (Li^+^: 980.0±388.3 mg; VPA: 1106.25±400.36 mg) and SGAs. Outcome measures: response rates, changes in YMRS, HDRS, and CGI-I	After 8 weeks, TPX+MS+SGA and bupropion+MS+SGA showed similar response rates (56% *vs.* 59%, respectively). Time to response ranged from 2 to 4 weeks. Both groups showed similar rates of ↓ from BL on the HDRS (20.5 to 10; 20 to 9.5 respectively), CGI-I, and YMRS (7 to 2; 8 to 2 respectively)	Both adjunctive TPX and bupropion were associated with reductions in depressive symptoms
45	60 BD, depressive; two groups: Paroxetine+MS (N=30; *$\bar{x}$*=47.1±15.2 yrs; 11 ♂; 19 ♀;23 BD-I, 7 BD-II); Venlafaxine+MS (N=30; *$\bar{x}$*=45.5±13.7 yrs; 9 ♂; 21 ♀;21 BD-I, 9 BD-II); HDRS>17	6-week RCT of add-on paroxetine (*$\bar{x}$*=32.3mg ± 11.2 or venlafaxine ($\bar{x}$=32.3mg ± 11.2) to MS (Li, 0.7 mg/L; VPA, 50 µg/ml; CBZ, 4µg/ml). Outcome measures: response, remission, switch rates.	After 6 weeks: Groups did not differ in HDRS ↓ (paroxetine+MS: -6.9; venlafaxine+ MS: -9.0); similar proportions of responders (paroxetine+MS: 50%; venlafaxine+MS: 59%); similar remission rates (paroxetine+MS: 37%; venlafaxine+MS: 41%); similar switch rates (paroxetine+MS: 3%; venlafaxine+MS: 13%)	Both venlafaxine and paroxetine are effective add-on treatments to MSs for bipolar depression. Switch rates, especially during treatment with venlafaxine, raise some concerns
46	318 BD aged 24-89 ($\bar{x}$=53.3±15.1; 41% ♂; 59% ♀)	Retrospective, naturalistic; Evaluation of anxiety comorbidity and response to MS (Li^+^ or AEs); remission criterion, no mood episodes for 2 years	24% with anxiety comorbidity; anxiety comorbidity associated with poorer response to AEs. No differences in response to Li^+^	Anxiety comorbidity ↓response to AEs
47	150 BDI, manic or mixed, aged 19 – 65; two groups (MS + RISP, N=75, median=37y, 32♂; 43♀); MS + Placebo, N=75, median 42y, 31♂; 44♀)); median:37y for MS + RISP group, 42y for MS + placebo group; 32♂; 43♀ for MS + RISP group, 31♂; 44♀ for MS + placebo group	Randomized, double blind, placebo controlled. MS + RISP (4mg/day) or MS + placebo x 3 weeks. Outcome measures: changes in YMRS at day 8 and endpoint (last available observation), % subjects showing 50% YMRS improvement, time to response (30% YMRS score reduction), CGI 1/2	Compared to BL, ↓ YMRS was significantly greater in the MS + RISP group (-10.2±1.1) than MS + Placebo group (-6.7±1.0) at week 1. At endpoint, 59% of pts. in the MS+ RISP group showed ≥50% ↓YMRS scores, compared with 41% in the MS + placebo group. 48% at week 1 and 61% at endpoint of the MS + RISP group scored 1 or 2 on the CGI, compared with 31% at week 1 and 43% at endpoint in the placebo group. Compared to BL, at week 1 and endpoint, BPRS ↓ was significantly greater in the MS+RISP group (-7.5±.09 and -10.1±1.1, respectively) than in the MS+placebo group (-3.8± 0.8 and -4.8± 1.1 respectively). At endpoint, the MS+RISP group had significantly greater improvement in the hostility and thought disturbance subscales of the BPRS than did the MS+placebo group	RISP, in association with MS, is more efficacious than placebo in the improvement in manic symptoms. Improvement in manic symptom is also rapid
48	BD aged 18-65 with at least one manic episode requiring hospitalization; DB study: 156 BD (80 ♂; 76 ♀); Open: 85 BD (39 ♂; 46 ♀)	3-week DB: MS+RISP, MS+placebo or MS+haloperidol; then 10-week open-label, add-on RISP; remission criterion, YMRS≤12	Greater remission in RISP or haloperidol group compared to placebo; 79% of pts. in remission after 10-week open-label treatment with RISP	RISP+MS combination is efficacious in manic episodes requiring hospitalization
49	22 BD with TRD. Two groups: pramipexole+MS (N=12; *$\bar{x}$*=40.9;±8.2%; 4♂; 8♀; 9 BD-I, 3BD-II; placebo+MS (N=10; *$\bar{x}$*=43.3±6.2; 7♂; 3*♀;* 6BD-I, 4BD-II); YMRS<12; HDRS >18	6-week, double-blind, placebo-controlled, single center trial of add-on pramipexole (1.0-2.5 mg/day) to MS (Li^+^ 1137.5±381.5 mg/day; VPA 916.7±129.1 mg/day; CBZ 400.0 ± 282.8 mg/day; LAM 283.3 ± 144.3 mg/day; GPT 450±212.1 mg/day). Outcome measures: ≥↓ 50% in HDRS scores; changes in CGI-S.	After 6 weeks, 67% of pts. on pramipexole+MS showed ≥↓ 50% on HDRS compared to 20% in the placebo+MS group. Mean change from baseline in HDRS was greater in pts. taking pramipexole+MS (48.0%±33.1) than for those taking placebo+MS (21.4%±36.3). Pramipexole+MS showed lower CGI-S (2.7±1.4) than placebo+MS (4.4±1.3). After 6 weeks, pramipexole+MS showed greater CGI-S score ↓ (-2.4±1.8) than placebo (-0.30±1.3)	Pramipexole is effective for TRD in BD.
50	45 BD, depressed, aged 18-75 yrs (*$\bar{x}$*=42.2±11.5; 30♂; 15*♀*; 30 BD-II; 15 BD-II); HDRS ≥10; YMRS≤12; GAF≤70	Open-label, add-on citalopram to MS (Li^+^, VPA, or CBZ). 8-week acute phase and for those who responded, 16 weeks continuation phase. Outcome measures: changes in YMRS and HDRS, occurrence of anger attacks (using modified AAQ) at 8 weeks; survival analyses at 8 and 16 weeks	At BL 38.6% have anger attacks, which dropped to 14.6% after 8 weeks. 73.3% of those having anger attacks at baseline did not have them after 8 weeks, whereas 8.7% of those who did not have anger attacks at baseline reported anger attacks after 8 weeks. Treatment response and rates of response to depression were unrelated. Survival analyses were similar between groups. Trait anger predicts anger attacks at BL and at week 8	Anger attacks in BD respond favorably to add-on citalopram and are better predicted by trait anger than hypomanic or depressive symptoms
51	99 BD-I aged 18-70, in remission from a manic/mixed episode; two groups: OLZ+MS (N=51; *$\bar{x}$*=43.5; 52.9%♂; 46.1%*♀)*; Placebo+MS (N=48; *$\bar{x}$*=39.0; 43.8% ♂; 55.2% *♀*); DSM-IV A and B criteria severity for current manic episode ≤3, no more than two B criteria, DSM-IV A criteria severity for current depression ≤3, no more than three A criteria	Double-masked placebo-controlled trial of add-on OLZ (5-20 mg) to MS (Li^+^, 954.6-1174.7 mg/day or VPA, 1060.4-1512 mg/day). Outcome measures: a) syndromic relapse: occurrence of DSM-IV manic, depressive, or mixed episode; b) symptomatic relapse: HDRS or YMRS≥15	Time to symptomatic relapse into either mania or depression was significantly longer for the combination group compared with the monotherapy group (163 days for OLZ+MS; 42 days for Placebo+MS). Women and white patients with OLZ+MS showed longer times to symptomatic relapse than pts with Placebo+MS (84 *vs.* 67 days, respectively)	OLZ+MS reduced relapse in BD episodes
52	909 BDI, manic or hypomanic, aged 16-60 ($\bar{x}$=35.1±13.7; 8♂; 10♀). YMRS>20 (for manics), YMRS>7 (for hypomanics)	Open label, add-on RISP to MS (Li^+^, VPA, CBZ, TPX), × 6 weeks; Outcome measures: reduction in YMRS, SARS, and CGI; response criterion (>50% reduction YMRS)	After 6 weeks, reduction of scores on the YMRS (from 32.9 ± 10.8 to 9.5 ±8.4) and CGI-severity (from 4.8 ± 1.1 to 2.1 ±.8) was significant. Response rates at week 6 were 70.7%. A higher reduction in the YMRS and CGI scores was found in the subgroup with psychotic features (24.2 ± 11.9; 2.9 ± 1.4 respectively) compared to the subgroup without psychotic features (22.6 ± 11.6; 2.5 ± 1.5 respectively)	RISP is effective for BD manic/hypomanic episode treatment
53	18 BDI, manic or hypomanic ($\bar{x}$=35.1 ± 13.7; 8♂; 10♀)	Open label, add-on QTP (mean 267.9±105.4 mg/day) × 4 weeks. Outcome measures: ↓ in YMRS, HDRS, BPRS and CGI; response criterion (>50% ↓ on the YMRS)	After 4 weeks, YMRS ↓ from 28.2 ± 7.6 to 9.3 ± 5.7; HDRS ↓ from 2.7 ± 2.4 to .9 ± 1; BPRS ↓ from 32.8 ± 11.2 to 15.8 ± 11.6; CGI ↓ from 5±0.8 to 2.3 ± .7. Response rates at week 4 were 72.2%	Add-on QTP is an effective treatment for manic/hypomanic episode of BD
54	59 UP-MDD, but scoring high on Angst's hypomania; good responder group: $\bar{x}$ age=47; 17% ♂; 83% ♀; poor responder group: $\bar{x}$ age=52; 19% ♂; 81% ♀	Retrospective, naturalistic, comparison between good and poor responders to MS augmentation on ADs; remission criterion, clinical judgment of treating psychiatrists	30% good responders, 70% poor responders; greater delay in prescribing MS augmentation and lower rate of MS in poor responders. No differences in hypomania and temperaments. Higher suicidal risk and agitation in poor responders	MS augmentation should be instituted without delay in patients with MDD meeting Angst's criteria for hypomania
55	479 BD aged 18-65 (215 ♂; 264 ♀)	Retrospective, naturalistic; BD discharged on MS, MS+FGA, MS+SGA; relapse criterion, rate and time to rehospitalization	No differences between groups in rehospitalization time and rate (23% BD discharged on MS, 27% MS+FGA, 25% MS+SGA)	Augmentation with antipsychotics does not improve mood stability
56	287 BD-I manic or mixed; aged 18-70 yrs; two groups: TPX+MS ($\bar{x}$=41.0±12.2; 58♂; 85*♀*); Placebo+MS ($\bar{x}$=39.0±11.9; 67♂; 77*♀*). YMRS ≥18	12-week, DB, placebo controlled, add-on TPX (50-400 mg/day) to MS (Li^+^, $\bar{x}$ serum level 0.7 mEq/l, or VPA, $\bar{x}$ serum level 70 µg/ml) and AP. Outcome measures: score change on YMRS, MADRS, CGI-S, BPRS, GAS	↓ YMRS scores in both TPX (-10.1±8.7) and placebo (-9.6±8.2) groups, with no between-group differences. CGI, BPRS, MADRS and GAS improved, without between-group differences	Add-on treatment with TPX is not superior to placebo in ↓ manic/mixed episodes
57	159 BD, depressive (*$\bar{x}$*=41.6±12.2; 83♂; 76*♀*; 115 BD-I, 42 BD-II). 228 acute AD trials, 111 AD continuation phase trials	Randomized, add-on bupropion ($\bar{x}$=286±132 mg/day), venlafaxine ($\bar{x}$=195±112 mg/day), and sertraline (286±132 mg/day) to MS (Li^+^, AE, AP). Acute phase: 10 weeks, those improved entered continuation phase (1 yr). If patients did not respond acutely to initial AD trial, they were randomly assigned to another AD. Outcome measures: response (CGI-BP=1 or 2; occurrence of 1) brief hypomania; 2) recurrent brief hypomania; 3) switch to full hypomania; 4) switch to mania	Acute phase: 48.7% of the trials reached response, that dropped to 32.5% after excluding those who had a switch. Switch rate to full (hypo)mania was 19.3%. 111 subjects reached sufficient response to enter the continuation phase. Continuation phase:67.8% trials showed AD response, but response rate in absence of a switch was 42.5%. 36.8% of trials switched to (hypo)mania. AD switch rates were not significantly different among the three ADs. In both acute and continuation phases, the threshold/subthreshold switch ratio was lowest with bupropion (acute: 0.85; continuation: 1.2), intermediate with sertraline (1.6 and 1.65, respectively) and higher with venlafaxine (3.6 and 3.75, respectively)	AD augmentation is not likely to yield a high rate of sustained AD response without a switch throughout both the acute and continuation treatment phases. Venlafaxine was associated with the highest relative risk of switch and bupropion with the lowest
58	10 drug-naïve BD-II aged 18-65 with monthly mood episodes	Randomized, DB, escitalopram 10 mg vs placebo for 9 months	Reduction in depression severity, percentage of impaired days in the escitalopram group	Results support usefulness of SSRIs in BD-II treatment
59	1127 BD-I after a recent manic or depressive episode (456 ♂; 671 ♀)	Open, LAM 100-200 mg/day+sedative/hypnotics vs LAM 100-200 mg/day+other psychotropics; stabilisation criterion, CGI≤3 for ≥4 weeks	Higher stabilization rates for LAM 100-200 mg/day+sedative/hypnotics vs other psychotropics	Adjunctive therapy with sedative/hypnotics may be useful in BD acute symptom control
60	55 BD-I ($\bar{x}$ age=35±12.8, 34 ♂; 21 ♀)	Retrospective, naturalistic, SGA monotherapy *vs.* SGAs+MS for 6 months; relapse criterion, recurrence of mood episode	Clinical improvement in both groups, no differences in relapse	SGAs can be useful in the long-term management of BD-I
61	10 BD-I outpts. (11–17 years) using a single MS and/or SGA, who had shown weight gain >5% of BL weight	Open. 11-week; medication switched to TPX during the first 4 weeks until 150 mg/day; YMRS main outcome to measure treatment response	Significant ↓in both YMRS score (F=10.21; *p*<0.01) and weight (F=8.04; p<0.01). $\bar{x}$ weight loss=2.62 kg at endpoint. 6/7 completers (85%) did not show symptom worsening on the YMRS after 11 weeks. Significant BMI ↓ from BL to endpoint (*p*=0.017). No increase in adverse events	TPX seems to have antimanic effects during the treatment maintenance phase associated with weight reductions
62	89 pregnant women ($\bar{x}$ age 32.7 years±5.4) BD-I (N=61) or BD-II (N=28)	Prospective observational study; Two groups based on MS status: 1) use of at least one MS at conception and continued ≥12 weeks of pregnancy; 2) MS discontinuation during 6 months before conception to 12 weeks of gestation. Follow up each trimester and at 6, 12, 24, and 52 weeks postpartum to ascertain recurrence of mania, hypomania (lasting ≥1 week), major depression, or a mixed state, and current treatments	During pregnancy, a total of 70.8% (63/89) of women experienced ≥1 episode of illness. Recurrence risk was 2.3 times greater after discontinuation of MS (53/62, 85.5%) than with continued treatment (10/27, 37.0%). Discontinuers spent >40% of pregnancy in an illness episode, *vs.* 8.8% of pregnancy of women continuing on MS. Median time to first recurrence was 9.0 (95% CI=8.0–13.0) weeks for discontinuers and >40 weeks (95% CI indeterminate) for continuers. Abrupt or rapid discontinuers (1–14 days; N=35) had 50% risk of recurrence within 2.0 (95% CI=1.0–6.0) weeks, gradual discontinuers (≥15 days, N=27) required 22.0 (95% CI=16.0–38.0) weeks to reach 50% recurrence risk (χ^2^= 25.9, df=1, p<0.0001). Excess of depressive-dysphoric polarity *vs.* manic-hypomanic episodes after discontinuation of MS (55/62 recurrences, 88.7%, versus 12/62, 19.3%, or 4.6-fold) compared to continued treatment (5/27, 18.5%, *vs.* 9/27, 33.3%, or 1.8-fold). Treatment-related risk factors, besides MS discontinuation, included: 1) polytherapy with two or more psychotropics (RR=2.3, *p*<0.001); 2) use of AD (RR=2.0, *p*<0.001); 3) primary MS other than Li^+^ (RR=1.6, p<0.001); 4) previous switch from depression to mania/hypomania during past AD treatment (RR=1.5, *p*<0.009); 5) abrupt MS discontinuation (RR=1.4, *p*=0.008). AD use and treatment discontinuation each operated independently as risk factors, even after adjusting for other indices of illness severity	Discontinuation during pregnancy of MS, particularly if abruptly, carries a high risk for new morbidity in women with BD, especially for early depressive and dysphoric states. However, this risk is reduced markedly by continued MS treatment
63	232, BD, euthymic, aged 22-79 ($\bar{x}$=52.2 ± 9.7; 81 ♂; 158 ♀, BD-I N=91; BD-II N=141). 6 groups: QTP monotherapy (N=41); Li^+^ monotherapy (N=39); VPA monotherapy (N=73); LAM monotherapy (N=31); QTP + Li^+^ (N=25); QTP + VPA (N=23)	Naturalistic, 4-yr follow-up. Mean QTP doses: monotherapy, 214 mg/day; QTP+Li^+^, 223.5 mg/day; QTP+VPA, 237.4 mg/day; LAM 72.2 mg/day. Mean plasma levels for Li^+^ or VPA, 0.7 mEq/l ± 0.2 for Li^+^ monotherapy, 0.7 mEq/l ± 0.1 for Li^+^+QTP, 52.1±17.2 ng/ml for VPA monotherapy, and 60.5 ng/ml ± 17.9 SD for VPA+QTP. Outcome measures: duration of euthymia/proportion of pts with no mood recurrences	After 4 years, pts. with Li^+^+QTP and Li^+^+VPA showed higher proportion of no mood episode (80% and 78.3%, respectively) than those with QTP alone (29.3%), Li^+^ alone (46.2%) and LAM alone (41.9%). Pts. with Li^+^+QTP and Li^+^+VPA did not relapse for longer times (41.4 and 39.2 months, respectively) than pts. on QTP (33.1 months) and VPA (30.1 months). Only pts. with Li^+^+QTP did not relapse for longer times than Li^+^ alone (33.1 months). Pts. with Li^+^ were superior to those with QTP in proportion of subjects without relapses and time spent without relapse	QTP as either monotherapy or combination therapy (with Li^+^ or VPA) has been found to be effective in preventing both major and sub-threshold depressive episodes
64	108 BD, euthymic, drug-free, ($\bar{x}$=52.2 ± 9.7; 43♂; 65 ♀, BD-I N=39; BD-II N=69). 3 groups: BD with early onset (<30y), middle onset (>30 and <45y), and late onset (>45y)	Naturalistic, 24-month follow-up. Drug free pts. received SGAs, Li^+^ or VPA. Outcome measure: relapse rates	After 24 months, $\bar{x}$ depressive relapses were less in the early-onset group ($\bar{x}$=0.66) than middle- ($\bar{x}$=1.37) and late-onset ($\bar{x}$=1.26)	MS treatment seemed to be more effective in preventing depressive episodes in early-onset BD pts. compared to middle- and late-onset pts. Middle- and late-onset BD pts. were similar
65	966 BD-I (open-label phase), with a recent depressive episode, aged ≥18 yrs.; 463 BD-I (randomization phase), aged ≥18 yrs.	First phase: open-label trial on LAM monotherapy (200-400 mg/day) for 16 weeks. Second phase: double-blind placebo- controlled trial on LAM monotherapy (200-400 mg/day). Outcome measures: occurrence of an intervention for manic/hypomanic/mixed symptoms, YMRS score ≥ 4, YMRS score ≥ 8, and YMRS score ≥ 14, survival analyses	Open-label phase: Compared to BL, YMRS ↑ by ≥14 points in 10% of pts., ↑ by ≥8 points in 20% of pts., by ≥4 points in 35% of pts. YMRS ↑ predicted by number of manic/hypomanic/mixed episodes in the preceding year. Randomized phase: no differences in % or HR of event occurrence between groups, LAM had consistently higher estimates of survival than placebo across all 4 thresholds of mania MRS scores at screening, and presence of ≥3 manic/hypomanic/mixed episodes in the preceding year significantly increased HR of reaching an event	LAM showed similar rates of manic relapse to placebo. During maintenance treatment, the likelihood of emergent manic or hypomanic features appears driven more by the pre-existing or historical burden of mania features, rather than the use of LAM
66	109 BD-II, depressive, aged 18-65 yrs (*$\bar{x}$* =40.3±11.5; 27♂; 82*♀*). CGI-BP-S depression score≥3 for >12 weeks on maintenance with Li^+^ or VPA+other medications (ADs and APs allowed)	Naturalistic, follow up (52 weeks) of add-on LAM (145.5±113.2 mg/day) to MS (Li^+^ or VPA) and APs (QTP, OLZ, ZIPR). Outcome measures: change in CGI-BP-S depression score	CGI-BP-S depression score ↓after 4 weeks of LAM add-on. Scores on the CGI-BP-S ↓ by about 1.8 points in the first 12 weeks and then remained stable. 49% completers. Completers and drop-outs differed for number of psychiatric hospitalizations (0.7±0.9 vs. 1.4±2.2) and history of suicide attempts (11.4% *vs.* 30.8%, respectively)	LAM is an effective add-on treatment for bipolar depression. Number of prior hospitalizations for depression and history of attempted suicide may be associated with poor response to adjunctive LAM treatment
67	23 adult BD inpts. (12 ♂; 11 ♀; 17 BD-I, 6 BD-II), HDRS ≥20	Randomized, DB, MS+citalopram 40 mg/day +ARP 10-30mg/day *vs.* MS+citalopram 40 mg/day+placebo for 6 weeks; remission criterion, HDRS ≤9	No differences between the two groups	Augmentation with ARP does not improve mood stability
68	50 BD, depressive pts., aged 18-70 ($\bar{x}$=439.7±10.3; 23 ♂; 31 ♀; 20 BD-I, 34 BD-II); MADRS>20 and YMRS<12	6 week – double-blind, placebo-controlled trial of VPA monotherapy (mean 1606±44 mg/day, range 1000–2000 mg/day). Outcome measure: improvement on MADRS, YMRS, CGI-BP, HARS. % pts. achieving response (≥50% ↓ in MADRS from BL)	VPA group improved more on the MADRS compared with placebo group at weeks 3, 4, 5, and 6 (mean change of MADRS total score for VPA over placebo=4.32). This is mainly driven by differences in the BDI subgroup. VPA group responded for 38.1% and remitted for 23.1%, placebo group improved and remitted by 10.7%	VPA is effective in treating bipolar depression in the BD-I subset
69	139 BD-I with manic or mixed episode after 6-week olanzapine *vs.* haloperidol *vs.* placebo DB trial. Monotherapy group (N=100): ($\bar{x}$=41.8; 41% ♂; 59% ♀); Combination group (N=39): ($\bar{x}$=43.2; 46% ♂; 54% ♀)	Open, 56-site (Japan) study; olanzapine 5-20 mg/day monotherapy switch from previous trial × 18 weeks, if lack of efficacy: olanzapine + MS (Li^+^, CBZ or VPA); safety assed by treatment-emergent adverse events; Remission criterion, YMRS≤12	Monotherapy group: 59% treatment-emergent adverse events, remission 93%; Combination group: treatment-emergent adverse events 79.5%, remission 61.5%	Results support efficacy of combination therapy of olanzapine + MS if olanzapine monotherapy lacks of efficacy
70	40 BD aged 24-84 yrs ($\bar{x}$=49±16; 10 ♂; 30 ***♀***; 21 BD-I, 19 BD-II) CGI-BP≥5	Open, add-on memantine 10-30 mg/day × 12 months; response/remission criterion, CGI-BP 1 or 2	After 6 months, 47.5% scored 1 and 25% 2; after 12 months, 52.5% 1 and 20% 2	Memantine helps overcoming resistance if added on ongoing treatment
71	13 BD, depressive, aged ≥18 yrs (6 ♂; 7 ♀; 12 BD-I, 1 BD-II). HDRS>15, YMRS>12.	8-week, open-label, add-on nefazodone 300-600 mg/day+MS or AP (Li^+^ 450- 600 mg/day; LAM 400 mg/day; VPA, 750-1250 mg/day; CBZ 400-600 mg/day, CLZ, 325 mg/day). Outcome measures: changes in HDRS and CGI-BP, remission, response	69% responded after 8 weeks, 31% remitted. HRDS ↓ from 26.1 ± 5.1 at BL to 18.5 ± 10.1 at week 8; CGI-BP ↓ from 24.2± 0.6 at BL to 3.4 ± 1.3 at week 8, both significant	The effectiveness of add-on nefazodone therapy is moderate
72	83 BD-I aged 18-65 (31 ♂; 52 ♀) with sleep disturbances, ≥5 Pittsburgh Sleep Quality Index	Randomized, DB, add-on ramelteon 8 mg × 24 weeks *vs.* placebo; relapse criterion, MADRS score ≥16 and/or YMRS≥15 or need of drug treatment	48.2% relapse. Ramelteon group less likely to relapse	Ramelteon has a potential utility in maintain mood stability
73	7423 PBD (N 3131 aged 6–12; N 4292 aged 13–18). Age $\bar{x}$ 12.73±3.38 on either MS (2479) or SGA (4944)	Retrospective cohort study. The outcome measures were psychiatric hospital admission, all cause medication discontinuation and treatment augmentation	Pts. who initiated on MS and SGA had comparable risk of psychiatric hospital admission (HR = 1.172, 95%CI: 0.827–1.660). Compared with those who initiated on MS, pts. who initiated on SGA were less likely to discontinue treatment (HR=0.634, 95% CI: 0.419–0.961) and less likely to receive treatment augmentation (HR=0.223, 95%CI: 0.103–0.484)	SGAs might be more effective and better tolerated than traditional MSs in BD maintenance treatment
74	23 euthymic BD (Age $\bar{x}$=48.13 yrs.±14.73; 16 ♂; 7 ♀)	Naturalistic, follow up. Outcome measures: mean time to recurrence after MS discontinuation	Median time of recurrence (all manic relapses) is 10 months. Total number of episodes and number of manic episodes negatively correlate with time to recurrence	Rates of relapse in the Indian population are similar to those present in western countries
75	3240 BD (1270 ♂; 1970 ♀) with no AD treatment during the previous year	Retrospective, naturalistic; AD monotherapy *vs.* AD+MS; switch to mania criterion, rate of mania 0-3 months, 3-9 months	↑ risk of switch was confined to pts. on AD monotherapy	AD monotherapy is associated with ↑ risk of mania
76	180 BD-I aged 18-65 (59♂; 121 ♀) in acute manic episode	Randomized, DB, add-on allopurinol 300 mg/die vs placebo × 6 weeks; response criterion, ≥50% ↓ in YMRS	No differences between groups in response	Results do not support add-on allopurinol as a treatment for acute mania
77	59 BD-II > 18 years old ($\bar{x}$ =4±12.5; 27 ♂; 32 ♀;) responded to treatment, HDRS ≤ 16	Randomized, DB, venlafaxine or Li^+^ monotherapy × 12 weeks, 6 additional months to evaluate relapse; response criterion, ≥50% ↓ in HDRS; relapse criterion, HDRS≥14+CGI≥4 for ≥14 days	67.7% venlafaxine versus 34.4% lithium subjects responded; no difference in relapse between treatment conditions during continuation monotherapy	Continuation venlafaxine and Li^+^ monotherapies provide similar prophylactic effectiveness
78	201 adult BD-I hospitalized for a manic episode (113 ♂; 88 ♀)	Retrospective, naturalistic; MS monotherapy, MS+SGA, MS+FGA; response criterion, 1-year rehospitalization	1-year rehospitalization rates lower in MS+SGA group (6.3%) compared to MS monotherapy group (24.3%) and to MS+FGA group (20.6%)	Results support efficacy of atypical antipsychotic adjunctive therapy to MS
79	Drug naïve BD aged 18-65 ($\bar{x}$ age= 38.8 yrs.; 56 ♂; 12 ♀) with mixed depression	Open, CBZ, Li^+^ or VPA monotherapy × 8 weeks; response criterion, ↓ ≥50% in HDRS+one mania scale (YMRS, CARS-M, BRMaS)	High agreement between the three mania rating scales; response on HDRS+YMRS=22.1%, on HDRS+BRMaS=20.6%, and on HDRS+CARS-M=23.5%	Results support the use of any scale to assess the efficacy of MSs in mixed depression; overall response is about 20%
80	344 BDI, depressive, aged 17-70 ($\bar{x}$=45.2±12.6; 78 ♂; 122 ♀). HDRS >18 and YMRS>8	8 week-DB, 67-cent3r (15 countries) RCT of add-on agomelatine (25-50 mg) on VPA or Li^+^. Additional 10-month continuation phase. Outcome measures: MADRS improvement; response criteria (MADRS improvement ≥50%); changes in HDRS, HARS, CGI, LSEQ, QLESQ	No differences. Results became significant (greater improvement in the agomelatine group *vs.* placebo) after excluding sites with pts. showing high placebo response	Agomelatine added on Li^+^ or VPA MS is ineffective for bipolar depression. Concerns for patient recruitment in some centers
81	159 BD-I aged ≥17 yrs. ($\bar{x}$=37.9±13.49; 79 ♂; 79 ♀). Patients remitted from recent manic episode on Li^+^ or VPA and RISP or OLZ add-on	Patients randomized to placebo substitution of RISP or OLZ at week 0 (*N*=52), after 25 weeks (*N*=54), and no substitution for 52 weeks (*N*=53) (endpoint). Outcome measures: Event rates (occurrence of any mood episode), time of any mood episode	After 52 weeks, event rate was higher in the 0-week group than in the 24- and 52-week groups. Time to any mood episode was longer in the 24- and 52-week groups compared to the 0-week group. No differences were found between 24- and 52-week groups. Time to mood episode was unchanged after considering subgroups taking OLZ. Instead, in those taking RISP, time to any mood episode in the 52-week group was similar to that in the 0-week group and shorter than in the 24-week group	Use of adjunctive SGAs to MS is beneficial for 24 weeks. However, these benefits are not apparent over 24 weeks. Relapse prevention by OLZ lasts longer than the one provided by RISP
82	80 BD aged 18-55 (23 ♂; 57♀; 65 BD-I, 15 BD-II)	Retrospective, naturalistic, treatment with Li^+^ and/or VPA for more than 2 years; response criterion, total Alda Scale score>5	34% good responders; no differences between Li^+^ and VPA groups. In the Li^+^+VPA group, psychotic, mixed, and atypical features associated with poorer response	Li^+^ and VPA show similar efficacy. Polypharmacy is associated with poor response
83	32 BD with subthreshold symptoms, aged 18-65 ($\bar{x}$=43.75*±*10.1; 12 ♂; 20 ♀; 21 BD-I, 11 BD-II). YMRS< 14 and/or MADRS>8 and <14	12-week, double blind-placebo controlled trial of QTP-XR, 300-600 mg, in addition to MS (Li^+^, VPA, LAM). Outcome measures: MADRS, HDRS-5, YMRS, CGI-BD score changes. Rates of remission, and early response: % of patients with HDRS<8 and YMRS<8, level of functioning (as assessed by FAST, GAF, EQ-5D, TOOL) and functional remission	The mean changes in MADRS total score from BL to week 6 were -2.44 in the QTP-XR and +2.50 in the placebo group. Changes in HDRS-5 at week 6 were -1.44 in the QTP-XR and +0.28 in the placebo group. At week 12, the QTP-XR group scored higher on the FAST-autonomy subdomain	QTP-XR is effective in treating subthreshold depressive symptoms
84	243 BDI, manic, aged 18-83 ($\bar{x}$=49.1±13.7; 107 ♂; 137 ♀)	Naturalistic, 34-center (Italy) 12-week follow-up study. Pts. starting/switching to AP and/or MS. Outcome measures: predictive factors of remission (YMRS ↓ ≥50%) and changes from baseline on YMRS, MADRS, FAST, CGI-BP	After 12 weeks, remission rate was 82.3%. No variables found to associate with remission. After 12 weeks, YMRS change was -22.0 ± 10.7. BL CGI-BP depression weakly predicted YMRS change. MADRS change was -6.1±8.2. BL YMRS weakly predicted MADRS change. CGI-mania and CGI-total score change was -6.1±8.2 and -2.7±1.6, respectively. BL YMRS scores weakly predicted CGI-mania and CGI-total score change. Mean FAST change was -17.4±17.3. BL YMRS scores predicted FAST score change	The initiated/changed pharmacological treatment for mania was associated with rapid improvement in manic symptoms and functioning. In contrast, the study has not clearly shown the association of any of the examined intrinsic and extrinsic factors with remission and clinical improvement
85	273 BD aged >18 ($\bar{x}$=40.8±11.07; 81 ♂; 192 ♀; 173 BD-I, 100 BD-II). Two trajectory-based groups: adherent (N=210) and nonadherent (N=63)	Naturalistic, 12-week follow-up. Outcome measures: factors differentiating adherent vs nonadherent group	The nonadherent group spent less time in euthymia (47%) than the adherent group (67.8%). Women more represented in the nonadherent (82.5%) than in the adherent group (66.7%)	Characteristics associated with belonging to the less adherent class were more time with symptoms (i.e., not euthymic), and female gender
86	413 youth BD aged 7-17.11; *N*=886 Li^+^, *N*= 1.752 MS	Naturalistic, longitudinal study. Data from the Course and Outcome of Bipolar Youth (COBY) study. Follow-up every 6 months over a mean follow-up of 10 years. “Lithium blocks”: Li^+^ for more than 75% of the follow-up weeks, regardless of other medications. “MS blocks”: MS but not Li^+^, for more than 75% of the time, i.e., antimanic AEs, FGAs and SGAs, and/or LAM. Clinical outcomes: suicide attempts and suicidal ideation; threshold and subthreshold depression; threshold and subthreshold (hypo)mania; psychosocial functioning; hospitalization; aggression; and SUD	During Li^+^ (*vs.* MS) follow-up periods, pts. were older, less likely to have lifetime anxiety, and less likely to be on AD (*p*<0.005). After covariate adjustment, the Li^+^ group (*vs.* MS) had half as many suicide attempts (*p*=0.03), fewer depressive symptoms (*p*=0.004), less psychosocial impairment (*p*=0.003), and less aggression (*p*=0.0004)	Li^+^ is associated with decreased suicidality, less depression, and better psychosocial functioning than MSs in a population of youths with BD
87	91 BD aged 18-70 ($\bar{x}$ age=41.6±14.2; 47 ♂; 44♀; 63 BD-I, 28 BD-II)	Retrospective, naturalistic, 2-site Campanian study; impaired glucose metabolism (*N*=49) *vs.* normal glucose metabolism (*N*=42); response to MS criterion, Alda scale score ≥7	Impaired glucose metabolism predicted low treatment response to MSs; in the impaired glucose metabolism sample, good response 4.4%, moderate response 23.1%, and poor response 22%; in the normal glucose metabolism sample, good response 17.6% (*p*=0.012), moderate response 23.1% (n.s.), and poor response 9.9% (*p*=0.001)	BD with impaired glucose metabolism are at risk for a poor response to MSs; poor metabolism predicts significantly less good response
88	16224 BD aged <65. Three treatment strategies: 6775 pts. (41.8%) MS (Li^+^, VPA, valpromide, CBZ, LAM) without SGA. 7268 pts. (44,8%) SGA (ARP, OLZ, RISP, QTP) without MS; 2181 pts. (13,4%) combination of MS+SGA	Historic cohort study using French national healthcare databases. Outcomes: treatment discontinuation, switch or addition, psychiatric hospitalization, suicide attempt, and death	The 1-year adjusted cumulative incidence of treatment failure was 75.7% (95%CI 74.9;76.3) in pts. using MS, 75.3% (74.6;76.0) in pts. using SGA, and 60.5% (58.3;62.6) in pts. with the combination. The adjusted difference in incidence of treatment failure for SGA compared with MS was -0.40% (-1.4;0.6 *p*=0.4) in the whole population, -2.2% (-3.3; -1.2 *p*<0.002) in pts. <65 years. Early discontinuation was the most often first occurring event over follow-up for all three treatment strategies. Treatment addition and discontinuation were slightly less frequent with SGA than with MS, while psychiatric hospitalization occurred more often in the SGA group. The 1-year adjusted cumulative incidence of partial discontinuation was 59.6% (95%CI 57.3;61.7) in pts. with combined treatment. The incidence of mortality was quite high as first event, particularly in the group receiving combinations. Treatment failure occurred in 64.8% (95%CI 64.0–65.6) of pts. with MS, 67.4% (95%CI 66.7;68.2) of pts. with SGAs, and 50.8% (95%CI 48.6;53.0) of pts. with a combination. The adjusted cumulative incidence of early treatment discontinuation was 37.0% (95%CI 36.1;37.8) with MS, 38% (95%CI 37.3;38) with SGAs, and 28.4% (95%CI 26.4;30.2) with a combination. Discontinuation was significantly more frequent with SGAs than with MSs with an adjusted difference in cumulative incidence=2.6% (95%CI 1.5;3.7; *p*<0.002)	The rate of treatment failure is very high in all age groups and for all treatment strategies. SGAs did not perform better than MSs in the whole study population and were even worse in the sensitivity analyses. SGAs are slightly more effective than MSs in younger pts., but less effective in older ones
88	3862 BD aged 65 and over. Three treatment strategies: MS: N 1450 (37.5%) SGA: N 2074 (53.7%) MS+SGA: N 338 (8.8%)	Historic cohort using French national healthcare databases. Outcomes: treatment discontinuation, switch or addition, psychiatric hospitalization, suicide attempt, and death	The adjusted difference in incidence of treatment failure for SGA compared with MS was +6.7% (4.1;9.1 *p*<0.002). Treatment addition was less frequent with SGA than with MS, while early discontinuation, psychiatric hospitalizations, and death were more frequent with SGA. When considering each type of outcome separately without stopping the follow-up when another type of outcome occurred, pts. with SGA (with or without MS) were at higher risk of death	SGAs are less effective in older pts. and fail more often than MSs in older pts. Mortality was particularly high in older pts. treated with SGA or a combination

## Discussion

Our aim in writing this review was to clarify which drug treatments achieve stabilization in the various phases of BD and across which age ranges or physiological conditions, like pregnancy and motherhood. Ideally, drug treatment strategies should have been tested phase-specifically in each age; however, studies were not sufficiently numerous for the childhood, older age and pregnancy, so the major focus will be on adulthood. Furthermore, rather than stabilization, the focus of most studies was on symptom improvement, so a reduction in depressive symptoms during the depressive phase and the reduction of excitement symptoms during manic/hypomanic phases are taken *per se* as stabilization, which *sensu strictu* are not.

### Adulthood

In this review, most results regarded adult patients in various phases of their disorder, so we will expose our findings according to the phase and the type of pharmacological treatment employed.

#### Manic/Hypomanic/Mixed

In a multicenter naturalistic study, Perugi et al. (84) investigated rates of remission and improvement in mood symptoms and functioning in manic patients treated with MSs and/or APs: Remission rates were 82% in 4 weeks, with Young Mania Rating Scale (YMRS) and Clinical Global Impressions-Bipolar (CGI-BP) mania scores rapidly decreasing. However, the authors did not identify any factors that were associated with remission. Although the extent of the occurrence of mixed states in BD is debated, it is estimated to be around 30% (89). In these cases, treatment should comprise judicious polytherapy (90).

##### Add-on SGAs

###### Risperidone

Acute treatment with add-on risperidone to MS (lithium [Li^+^], valproate and carbamazepine) has been investigated in five studies, which include one naturalistic (39), two placebo-controlled (42, 47), and two-open label studies (36, 51). Add-on risperidone (4 mg/day) has proven to be superior to placebo in decreasing manic/mixed symptoms as shown by greater reduction of YMRS and Clinical Global Impressions (CGI) scores. In two separate open-label studies, add-on risperidone (3-4 mg/day) showed similar rates of response after 4 and 6 weeks (74% and 70% respectively), while euthymia after 6 months was present in 73% of patients. Add-on risperidone (2-6 mg/day) to MS failed to show superior antimanic effects than add-on haloperidol (range: 4-12 mg/day) in one study (42), whereas in another study of inpatients, it showed to be superior to add-on SGAs in reducing mania at discharge (39). In the same study, no differences in effectiveness/efficacy were found as compared with add-on olanzapine (5-20 mg/day).

###### Olanzapine

Acute effectiveness/efficacy of olanzapine was investigated in one double-blind, placebo-controlled trial (43). Add-on olanzapine treatment (5-20 mg/day) to patients previously treated with MSs (Li^+^ or valproate) was superior to placebo in reducing YMRS scores and was associated with higher rates of remission. Furthermore, olanzapine monotherapy was similarly effective independently from whether the patients had failed or succeeded in the past to respond to another MS for mania.

###### Quetiapine

Effectiveness of add-on quetiapine (mean 267.9 ± 105.4 mg/day) to a MS in reducing manic/mixed symptoms was investigated by one open-label trial (53). After four weeks, add-on quetiapine reduced both manic and depressive symptoms, as demonstrated by the reduction of both YMRS and Hamilton Depression Rating Scale (HDRS) scores.

##### Mood-stabilizers

Add-on MS (Li^+^, carbamazepine, and valproate) to olanzapine (5-20 mg) in manic/mixed patients was associated with greater remission rates (61% *vs.* 95%) than olanzapine monotherapy (69). On the other hand, Goldberg et al. (32) showed that patients with Li^+^ or AE monotherapy had similar response rates and duration of remission, whereas time to remission in cases of combined therapy is somehow longer. This outcome may be related to the fact that people receiving combined treatment added the antiepileptic after an ineffective Li^+^ trial. After starting the combined therapy, time to remission was similar to the monotherapy group, i.e., 2-3 weeks.

Gabapentin (900-1200 mg/day) add-on treatment to antimanic drugs (Li^+^, valproate, and risperidone) was effective in rapidly reducing HDRS and Bech-Rafaelsen Mania Scale (BRMaS) scores after 1 week. In the following month, BRMaS scores stabilized, whereas HDRS scores continued to decrease (34). On the other hand, topiramate (50-400 mg/day) added on a MS (Li^+^, valproate) or an AP failed to show superior efficacy than placebo add on after 12-weeks (56).

##### Other

Add-on memantine in not stabilized BD is related to 47.5% and 52.5% rates of remission after 6 and 12 months (70), whereas sedatives (mainly BDZs) added on lamotrigine monotherapy (100-200 mg/day) in patients with either manic or depressive episode, were associated with higher rates of stabilization than adding other psychotropic drugs (mainly SGAs, ADs, and MSs) (59). Add-on nutritional supplements, like vitamins and minerals, proved able to reduce in some patients both manic and depressive symptoms (38), whereas allopurinol was not superior to placebo in reducing manic symptoms (76). However, allopurinol also improved YMRS scores in its double-blind study and with a greater effect size than what vitamins and chelated minerals were able to achieve in the open study.

Summarizing the evidence of studies treating acute episodes of mania or mixed, adding one SGA to a MS seems the best strategy to stabilize mood. The evidence of the antimanic effect of SGAs are most prominent for risperidone, a bit less for olanzapine and quetiapine. There are no differences between olanzapine and risperidone or valproate and Li^+^ as regards their antimanic effect. The evidence of differences between SGAs and FGAs as for their antimanic effect is at least poor and conflicting.

#### Depression

##### Add-on SGAs

Two double-blind placebo-controlled trials evaluated the effectiveness of aripiprazole (67) and quetiapine (83) as add-on treatments for bipolar depression. Quante et al. (67) failed to demonstrate superiority of augmentation therapy with aripiprazole (10-30 mg/day) as compared to placebo in patients treated with citalopram (40 mg/day) and a MS. Conversely, Garriga et al., showed that add-on quetiapine-extended release (300-600 mg/day) to a MS (Li^+^, valproate, or carbamazepine) was superior to placebo in improving subthreshold depressive symptoms after 6 weeks, and also in improving functioning after 12 weeks.

##### MSs

Hantouche et al. (54) assessed the characteristics of poor *vs.* good responders to add-on MSs (Li^+^, carbamazepine, and valproate) treatment to ADs in major depressive disorder patients with depression who met Angst's criteria for lifetime presence of subtle hypomanic and cyclothymic features, i.e., patients that the authors consider as belonging to the bipolar spectrum. Poor responders were prescribed a MS later than good responders, suggesting that MS augmentation should be undertaken without delay.

###### Valproate Monotherapy

Two studies investigated the effectiveness of valproate monotherapy in relieving depressive symptoms (41, 68). Valproate was associated with a 63% response after one year in patients with BD-II. Valproate monotherapy was also superior to placebo in improving HDRS scores after 3, 4, 5, and 6 weeks. However, differently from Winsberg et al. (41), such difference in Muzina et al. (68) was mainly driven by data regarding the subgroup affected by BD-I.

###### Lamotrigine

Two studies investigated the effectiveness/efficacy of lamotrigine in BD depression, either as monotherapy (65), or as add-on treatment (66). After 16 weeks, lamotrigine monotherapy (200-400 mg/day) increased YMRS scores by more than 4 points in 35% of patients, and such increase was predicted by the number of manic/hypomanic/mixed episodes in the preceding year. lamotrigine add-on treatment (145.5 ± 113.2 mg/day) to a MS (Li^+^ or valproate) or APs (quetiapine, olanzapine, or ziprasidone) reduced CGI-BP-S scores after 4 and 12 weeks. Such scores remained significantly lower during the following year, indicating successful stabilization.

###### Topiramate

Effectiveness of add-on topiramate (50-100 mg/day) in reducing both manic and depressive symptoms and in inducing response was compared with add-on bupropion (100-400 mg/day) to a MS (Li^+^ or valproate) and SGAs. Add-on treatments with either topiramate or bupropion were able to induce similar response rates (56% *vs.* 59%, respectively), within a similar time lag (2-4 weeks). Reductions in YMRS, HDRS, and CGI-I scores were also similar.

##### ADs

Bottlender et al. (37) evaluated the impact of AD treatment on the incidence of switches from depression to mania/hypomania in 158 BD-I patients with depression. Rates of switches were 25%, with higher risks for patients taking tricyclic antidepressants (TCAs) and lower for those on combined AD+MS treatment.

###### Add-on paroxetine

Three studies evaluated the effectiveness/efficacy of paroxetine in reducing depressive symptoms and rates of switch. Young et al. (35) compared the effectiveness/switch rates of either add-on paroxetine (36 mg/day) or additional MS (Li^+^, 1300 mg/day or valproate 1200 mg/day) to stable MS treatment. Both add-on treatments were associated with significant reductions in HDRS scores after 6 weeks, with no significant YMRS score increases.

Vieta et al. (45) compared add-on treatment with paroxetine (32.3 mg ± 11.2) or venlafaxine (179.2mg ± 91.0) to a MS (Li^+^, valproate, or carbamazepine) and investigated response, remission, and switch rates. After 6 weeks, similar proportions of responders (paroxetine+MS: 50%; venlafaxine+MS: 59%) and similar remission rates (paroxetine+MS: 37%; venlafaxine+MS: 41%) were found. Venlafaxine showed higher, even though not significantly so, rates of remission (48% *vs.* 43% with paroxetine). Nevertheless, the authors concluded that acute add-on treatment with venlafaxine raises concerns due to the higher rates of switch, although rates did not differ significantly, but only numerically (13% with venlafaxine, 3% with paroxetine). Authors stressed the need to replicate their preliminary findings, but no follow-up ensued.

###### Venlafaxine, Sertraline, and Bupropion

Amsterdam et al. (77) showed superiority of venlafaxine monotherapy over placebo in BD-II patients as concerns response rates at the 12-week endpoint (67.7% *vs.* 34.4%, respectively). Two randomized trials investigated the effectiveness/switch rates of add-on venlafaxine, sertraline or bupropion to MSs (Li^+^, valproate, or carbamazepine). Post et al. (40) reported a 37% response rate after 10 weeks, with 14% of switches into mania/hypomania, On the other hand, Leverich et al. (57) showed that after 10 weeks, response rates were 48.7%. However, response rates dropped to 32.5% after excluding patients who had a switch. Switch rate to full (hypo)mania was 19.3%, with higher rates for venlafaxine and lowest for bupropion. Both studies showed that AD augmentation is not likely to yield a high rate of sustained AD response without a switch.

###### Escitalopram

The study of Parker et al. (58) showed superiority of 10 mg of escitalopram monotherapy over placebo in a double-blind crossover study lasting 9 months, in reducing symptom severity and percent days impaired in a small sample of 10 drug-naïve patients with BD-II and monthly mood episodes.

###### Other

Goldberg et al. (49) evaluated the effectiveness of add-on pramipexole (1.0-2.5 mg/day) to MSs (Li^+^, valproate, or carbamazepine) in improving HDRS and CGI-S scores. After 6 weeks, pramipexole was superior to placebo in reducing depressive symptoms (pramipexole+MS was followed by more than 50% drop in HDRS scores compared to 20% in the placebo+MS; furthermore, it was associated with lower CGI-S scores (2.7 ± 1.4) than placebo+MS (4.4 ± 1.3). On the other hand, add-on therapy with agomelatine (25-50 mg) to a MS (Li^+^ and valproate) was not superior to add-on treatment with placebo in reducing depression after8 weeks (80). Goldberg et al. (71) found moderate antidepressant effect of nefazodone (300-600 mg/day) added on a MS (Li^+^, lamotrigine, valproate or carbamazepine) or an AP (clozapine).

Concluding, in the acute treatment of depression, adding ADs on ongoing MS treatment is effective in improving mood symptoms but it is also related to an increase in switch rates, specifically in BD-I or mixed samples. The evidence points to higher switch rates during add-on treatment with venlafaxine, a drug that inhibits the reuptake of both norepinephrine and serotonin, or TCAs, a group of drugs that are effective in blocking both transporters similarly to venlafaxine, than with selective serotonin reuptake inhibitors (SSRIs) or bupropion, which blocks the reuptake of norepinephrine and dopamine, and leaves the serotonin transporter almost unaffected. Risk of switch seems intermediate for SSRIs and lower with bupropion. ADs are effective in the short-term treatment of BD-II, even in monotherapy, but switch rates are not clearly evaluated across studies. Monotherapy with valproate and lamotrigine showed also short-term effectiveness, like topiramate and quetiapine-extended release add.

#### Long-Term Studies

##### APs

Two retrospective naturalistic studies investigated rates of relapse over a 1-year period in patients with BD (59, 78) treated with MS monotherapy, MS+SGAs and MS+FGAs, and reported conflicting results. Rehospitalization rates have been reported not to differ after a 1-year follow-up (Patel et al., 2006) or to be lower in patients receiving MS+SGAs, compared to MS monotherapy and MS+FGAs (78). Differences in sample characteristics [BD-I in Patel et al. [2006] and BD-I/BD-II mixed sample in Hochman et al. (78)] or type of SGA used might have played a role in such discrepancy. In partial agreement with Patel et al. (55), Tournier et al. (88), found similar treatment discontinuation rates, i.e., > 60% across the aforementioned three groups during a 1-year period, with slightly, but not significantly lower rates in the MS+SGAs combined group than the other two. Bernardo Dell'Osso et al. (64) investigated relapse rates after over 2 years in patients with early, middle, and late onset of BD, and found that MS treatment (Li^+^ or valproate+SGAs) are more effective in preventing depressive episodes in those patients with an early BD onset.

###### Olanzapine, Risperidone, and Quietiapine

Two placebo-controlled trials (51, 80) and one naturalistic study (63) investigated the effectiveness in relapse prevention of olanzapine, risperidone and quetiapine. Tohen et al. (41) found that patients on combined olanzapine (5-20 mg)/MS (Li^+^, 954.6-1174.7 mg/day or valproate, 1060.4-1512 mg/day) treatment had a longer mean time to symptomatic relapse into mania or depression then patients receiving MS+placebo (163 and 42 days, respectively). The effectiveness of add-on olanzapine was superior than add-on placebo or add-on risperidone in increasing the time of syndromic relapse during the short- (24 weeks), but not in the long-term (81). Altamura et al. (63) investigated rates of relapse over 4 years in patients treated with Li^+^ or valproate or lamotrigine or quetiapine as monotherapy or a combination of quetiapine to either Li^+^ or valproate. Patients with a combined treatment (quetiapine+Li^+^ and quetiapine+valproate) showed higher rates of euthymia (80% and 78.3%, respectively) than those with quetiapine alone (29.3%), Li^+^ alone (46.2%) and lamotrigine alone (41.9%). Patients with Li^+^+quetiapine and Li^+^+valproate did not relapse for longer times (41.4 and 39.2 months, respectively) than patients on quetiapine (24.9 months) and valproate (26.3 months) alone. Only patients with Li^+^+quetiapine did not relapse for significantly longer times than Li^+^ alone (33.1 months). Furthermore, patients with Li^+^ monotherapy showed smaller relapse rates than those with quetiapine monotherapy.

##### MSs

DePaulo et al. (31) investigated self-reported mood stability in patients with BD on long-term lithium therapy and found greater ratings of absence of mood swings than HCs. Ahn et al. (82) found that treatment response rates did not differ among patients with add-on Li^+^ to SGAs (quetiapine, olanzapine, risperidone, aripiprazole, paliperidone, clozapine, amisulpride), or other MSs (lamotrigine and carbamazepine) as compared to those receiving add-on treatment with valproate. On the other hand, Savas et al. (60) found that adjunctive therapy with MSs (Li^+^, valproate, carbamazepine or lamotrigine) to SGAs (risperidone, olanzapine or quetiapine) was not superior in preventing relapses as compared with SGAs alone over a 6 month-period.

Mean time of relapse after MS discontinuation was investigated by Sharma et al. (74) in a sample of Indian patients. Mean time to relapse was 10 months, and all relapses were manic, thus replicating existing data in samples belonging to Western countries. Steardo et al. (87) showed that impaired glucose metabolism was associated to poor long-term response to MSs (Li, valproate, lamotrigine, and carbamazepine) and APs. On the other hand, Henry et al. (46) showed that anxiety was a predictor of poor long-term (2 years) response to AEs, but not to Li^+^.

One open label, placebo-controlled trial (65) tested the effectiveness of lamotrigine monotherapy (100-200 mg/day) in reducing switch rates over 6 months. The authors found no differences between lamotrigine and placebo in percentage or hazard ratio for a medical intervention due to the onset of a mood episode. However, patients on lamotrigine monotherapy had consistently higher survival estimates than patients on placebo. Furthermore, YMRS scores at screening and presence of ≥3 manic/hypomanic/mixed episodes in the preceding year significantly increased the hazard ratio for a mood episode. The authors concluded that emergent manic or hypomanic features appear to be driven by the pre-existing or historical burden of mania features, rather than the use of lamotrigine.

##### ADs

Amsterdam et al. (77) found no difference in relapse rates over 6 months between patients with BD-II treated with venlafaxine or with lithium monotherapy. Two studies evaluated manic switch rates over one year of add-on bupropion, venlafaxine or sertraline to MS in patients who had responded in the past to AD augmentation (40, 57). In both studies, switch rates were higher than 30% (33% and 36%). AD switch rates were not significantly different among the three ADs; however, the threshold/subthreshold switch ratio was lowest with bupropion (1.2), intermediate with sertraline (1.65), and highest with venlafaxine (3.75). The long-term administration of 25-50 mg/day of the strong melatonin MT_1_ and MT_2_ receptor agonist and moderate serotonin 5-HT_2C_ and 5-HT_2B_ receptor antagonist, agomelatine, as an add-on to a MS, did not result in different switch rates compared to placebo added on a MS (80). However, also the response rates were similar in the two groups, raising questions about the antidepressant potency of agomelatine.

##### Other

Norris et al. (72) investigated the long-term effectiveness of add-on ramelteon (8 mg/day), a sleep inducer which shares with agomelatine the strong melatonin MT_1_ and MT_2_ receptor agonist activity, and is also endowed with weak MT_3_ and 5-HT_2B_ activity, to standard medications (including APs, MSs, ADs, and stimulants) in stabilized patients with BD over a 6-month period. As compared with placebo, patients with add-on ramelteon showed lower rates of relapse into any mood episode than placebo.

Studies focusing on stabilization of adult patients make the greatest part of those included in this review. As far, results appear to be inconsistent if not conflicting, but there is weak evidence supporting either the addition of a SGA to MS or using a SGA alone, which both confer mood stabilization that is superior to that obtained using MSs alone, at least in the medium term. For timeframes extending over six months, results are more conflicting. However, evidence supporting the effectiveness of combined therapy in reducing relapse is stronger than the one supporting the superiority of the use of MS or SGA alone. Li^+^ seems not to be superior to valproate in stabilizing mood as an add-on treatment. Add-on ADs to MSs are related with higher switch rates. If this holds true for BD-I or mixed samples, there is a week, preliminary evidence that this might not be true for BD-II.

### Children/Adolescents

Tramontina et al. (61) showed that the switch to a monotherapy with topiramate (150 mg/day) in youth (11-17 years) with BD, previously treated with MSs (Li^+^, valproate) or SGAs (risperidone), was associated to both reduction of YMRS scores and weight loss after 4 weeks.

Chen et al. (73) retrospectively investigated relapse rates after 12 months of treatment with either MSs (Li^+^, valproate, or carbamazepine) or SGAs (risperidone, aripiprazole or quetiapine). Patients who initiated MSs and SGAs had a comparable risk of psychiatric hospital admission; however, patients who initiated on SGAs were less likely to discontinue treatment and less likely to receive treatment augmentation. Hence, the authors concluded that in youths with BD, SGAs might be more effective and better tolerated than traditional MSs as a maintenance treatment. Conversely, Hafeman et al. (86) investigated suicide attempts and suicidal ideation, rates of threshold and subthreshold depression, (hypo)mania, psychosocial functioning, hospitalization, aggression, and substance use disorders in patients receiving Li^+^ or medications other than Li^+^ (AEs, FGAs and SGAs), regardless of other psychotropic medications, for more than 75% of the 10-year follow up. They found that Li^+^-treated youths were less likely to have lifetime anxiety, and less likely to be on ADs. Youth on Li^+^ had half as many suicide attempts, fewer depressive symptoms, psychosocial impairment due to illness, and less aggression than those not treated with Li^+^.

### Elderly

Sanderson et al. (33) compared length of stay and symptom improvement in elderly inpatients receiving monotherapy with Li^+^, valproate, or carbamazepine and found no significant differences across the groups. Tournier et al. (88) investigated rates of treatment discontinuation, switch, adjunctive medication, hospitalization, suicide attempt, and death over a 1-year period in patients treated with either MS (Li^+^, valproate, carbamazepine, and lamotrigine), SGAs (risperidone, aripiprazole, quetiapine, and olanzapine) or a combination of the two classes. Treatment failure was higher in those receiving SGAs than MSs. Addition of another drug was less frequent in those taking SGAs than in those taking MSs, while early discontinuation, psychiatric hospitalizations and death occurred more frequent in patients who were prescribed SGAs. The authors concluded that, in older patients, SGAs are less effective and fail more often than MSs. Mortality was particularly high in SGA-treated elderly patients, either as a monotherapy or in combination with MSs.

### Pregnancy

Viguera et al. (62) studied 89 pregnant women with polytherapy (including MSs, ADs, APs) for BD who 1) used at least one MS (Li^+^, valproate, carbamazepine, gabapentin, lamotrigine) or AP (olanzapine and quetiapine) at conception and continued treatment for more than 12 weeks; 2) discontinued MSs during the 6 months preceding the conception and for the following 12 weeks. Pregnant women were followed up each trimester and at 6, 12, 24, and 52 weeks postpartum to ascertain recurrence of mania, hypomania (lasting ≥1 week), major depression, or a mixed state, and current treatments. The authors found that 70.8% of women experienced ≥1 episode during pregnancy. Risk of recurrence was 2.3 times higher in those who discontinued treatment than in those who continued (85.5% *vs.* 37.0%, respectively). Discontinuers spent >40% of pregnancy in an illness episode, *vs.* 8.8% of pregnancy of women continuing on MS. Median time to first recurrence was 9.0 weeks for discontinuers and >40 weeks for continuers. Those who abruptly or rapidly discontinued MS treatment (< 14 days) had 50% risk of recurrence within 2 weeks, whereas gradual discontinuers (> 14 days) required 22 weeks to reach 50% risk of recurrence. Treatment-related risk factors, besides MS discontinuation, included polytherapy with two or more psychotropic drugs, use of ADs, primary MS other than Li^+^, and previous switch from depression to mania/hypomania during past AD treatment.

### Final considerations

Stabilizing treatments through the lifespan differ. In youth, SGAs are more tolerated and effective than MS in stabilizing mood (73). However, Li^+^ remains the cornerstone of mood stabilization as seeb in pediatric populations with BD, as it protects from impulsive acts and suicidal behavior (86). Furthermore, Li^+^ is also important for dimensions related to impulsive behavior and mood dysregulation, which are often encountered in such population (91). In adults, the use of add-on SGAs to MS in the treatment of manic/mixed state is still important, at least in the first half year of treatment. The combined treatment seems to confer greater mood stabilization. There is also preliminary evidence for greater effectiveness of some SGAs, like olanzapine, quetiapine, and risperidone, compared to MS monotherapy, but confirmatory studies are needed. In the elderly, the use of SGAs is contraindicated because of the impact on health and higher risk of death (all APs have a warning for increased risk of stroke in the elderly). Henceforth, the ratio of SGA/MS use varies across the lifespan, being highest during youth (frequent use for longer times of SGAs), intermediate in adult life (combined therapy), and low in the elderly (greater use of MSs).

#### Limitations

We based our conclusions on findings of sometimes underpowered studies, conducted with no double blinding, and often conducted on small samples. There is temporal discontinuity in the included studies, in that earlier years are less densely represented than recent years, and this might have affected the relative quality of the included studies. However, we found that most pre-millennial studies to be of high quality in both design and performance whereas not all recent trends in article standards resulted in improved data. The ways in which mood stabilization was considered and measured differed among studies. Only one study asked participants to rate their mood on a continuous visual scale, most others measured it as a reduction in HDRS or YMRS scores. This affected the evaluation of the stabilizing effect of the drug tested. Generally, we could not meta-analyze the eligible studies due to their extreme methodological differences in both design and assessment of outcomes; for example, about half were open-label and the other half double-blind. Furthermore, many were sponsored by the pharmaceutical industry, raising concerns that they could be biased in some sense. Risk of bias was high in most studies. Another limitation was that we did not assess the effects of physical therapies, like electroconvulsive therapy, deep and repetitive transcranial magnetic stimulation, and direct current transcranial stimulation, that may play a part in BD patients' treatment (92-94), but this would go beyond the scope of this review.

Summarizing, the indications for different treatments across the lifespan in BD are not supported by sufficient evidence, but appear nevertheless to differ. This is due to the dearth of studies carried out heretofore. The need for the future is for studies following the same methodology and adopting a consensus definition of stabilization.

## Conclusions

Mood stabilization is currently achieved at suboptimal levels. The evidence gathered heretofore is quite insufficient to propose treatment recommendations for cAdolescents, pregnant women, and elderly people. Regarding adults, in manic/mixed phases AP drugs, especially SGAs, have shown usefulness in acute to medium term treatment, especially in combination with MSs. The latter, especially Li^+^, is still the mainstay of chronic treatment, even though there is increasing evidence supporting the superiority of long-term combined therapy. Depressive phases of BD benefit from MS and quetiapine treatment, and there is some concern with the switch-inducing potential of some ADs, but less with others. The use of ADs in bipolar depression is safer when the AD is prescribed along with a MS.

## Data Availability Statement

All datasets generated for this study are included in the article/supplementary material.

## Author Contributions

GS and AS designed the review. All authors were involved in selection of eligible material and in Delphi rounds to reach consensus. AS and GK wrote the introduction, methods and results, designed the search strategy, gathered eligible material, and supervised the writing of the paper along with LJ and GS. GS, AK, DJ, and LD wrote the discussion. GK and AS wrote the limitations and conclusions. All authors approved the final form of the document. AS and AK equally contributed to the writing of the manuscript.

## Conflict of Interest

The authors declare that the research was conducted in the absence of any commercial or financial relationships that could be construed as a potential conflict of interest.

## Abbreviations

AAQ, Anger Attacks Questionnaire; AD(s), antidepressant drug(s); AE(s), anticonvulsant(s), antiepileptic(s); AP(s), antipsychotic(s); APT, adjunctive personalized treatment; ARP, aripiprazole; BD, bipolar disorder; -I, type I; -II, type II; BL, baseline; BPRS, Brief Psychiatric Rating Scale; BRMaS, Bech-Rafaelsen Mania Scale; CARS-M, Clinician-Administered Rating Scale for Mania; CBZ, carbamazepine; CGI, Clinical Global Impressions; -S, severity; -I, improvement; CGI-BP, Clinical Global Impressions Overall Scale for Bipolar Disorder; CLZ, clozapine; DB, double-blind; EQ-5D, the quality of life questionnaire in relation to health status “Euroqol”; FAST, Functioning Assessment Short Test; FGA(s), first-generation antipsychotic drug(s); GAF, Global Assessment of Functioning; GAS, Global Assessment Scale; GPT, gabapentin; HAL, haloperidol; HARS, Hamilton Anxiety Rating Scale; HDRS, Hamilton Depression Rating Scale (-N, item number specifying version); HCs, healthy controls; HDRS, Hamilton Depression Rating Scale; HR, hazard ratio; IDS, Inventory of Depressive Symptomatology; LAM, lamotrigine; LSEQ, Leeds Sleep Evaluation Questionnaire; Li^+^, lithium; MADRS, Montgomery-Åsberg Depression Rating Scale; MDD, major depressive disorder; MS, mood stabilizer; NOS, not otherwise specified; n.s., not significant; OLZ, olanzapine; OPT, optimized personalized treatment; PANSS, Positive And Negative Syndrome Scale; pts., patients; QLESQ, Quality of Life Enjoyment and Satisfaction Questionnaire Short Form, QTP, quetiapine (-XR, extended-release), RISP, risperidone; SABT, schizoaffective-bipolar type; SCZ, schizophrenia spectrum disorders; SGA(s), second-generation antipsychotic drug(s); SUD, substance use disorder; TCAs, tricyclic antidepressants; TOOL, the TOlerability and quality Of Life; TPX, topiramate; TRD, treatment-resistant depression; VPA, valproate; YMRS, Young Mania Rating Scale; yrs, years; ZIPR: ziprasidone; $\bar{x}$, mean; ♂, male; ***♀***, female; ±, standard deviation; , titrated up to or went from to; ↑, increase(d); ↓, decrease(d), drop.
